# Retraction Note: Ulnar malignant peripheral nerve sheath tumour diagnosis in a mixed-breed dog as a model to study human: histologic, immunohistochemical, and clinicopathologic study

**DOI:** 10.1186/s13000-016-0570-7

**Published:** 2016-11-02

**Authors:** Abbas Tavasoly, Javad Javanbakht, Fariba Khaki, Ehsan Hosseini, Alimohammad Bahrami, Mehdi Aghamohammad Hassan, Mohammadmehdi Mirabad

**Affiliations:** 1 Department of Pathology, Faculty of Veterinary Medicines, Tehran University, Tehran, Iran; 2 Paraveterinary Faculty of Ilam, University of Ilam, Ilam, Iran; 3 Department of Clinical Science, Faculty of Veterinary Medicine, Tehran University, Tehran, Iran

The Editor-in-Chief and Publisher have retracted this article [1] because the scientific integrity of the content cannot be guaranteed. An investigation by the Publisher found it to be one of a group of articles we have identified as showing evidence suggestive of attempts to subvert the peer review and publication system to inappropriately obtain or allocate authorship. This article showed evidence of plagiarism (most notably from the articles cited [2–6]) and peer review and authorship manipulation.
